# Early‐Phase Computed Tomography Bowel Diameter and Neurological Outcomes Post‐Extracorporeal Cardiopulmonary Resuscitation

**DOI:** 10.1155/emmi/4291375

**Published:** 2026-10-09

**Authors:** Shunki Hiyama, Mizuki Inoue, Ken Inoue, Taichi Kato, Keita Shibahashi, Kazuhiro Sugiyama

**Affiliations:** ^1^ Tertiary Emergency Medical Center, Tokyo Metropolitan Bokutoh Hospital, 4-23-15 Kotobashi Sumida-ku, Tokyo 130-8575, Japan, metro.tokyo.jp

**Keywords:** diagnostic imaging, extracorporeal membrane oxygenation, out-of-hospital cardiac arrest, prognosis, return of spontaneous circulation

## Abstract

**Aim:**

We aimed to conduct an exploratory investigation on the association between bowel diameter on early‐phase abdominal computed tomography (CT) and neurological outcomes in patients undergoing extracorporeal cardiopulmonary resuscitation (ECPR) for out‐of‐hospital cardiac arrest (OHCA).

**Methods:**

This observational study included patients who underwent ECPR for OHCA and abdominal CT before intensive care unit admission between January 2015 and December 2021 at a tertiary emergency medical center. Maximum bowel diameter was measured with a picture archiving and communication system by an investigator blinded to outcomes. The primary endpoint was unfavorable neurological outcomes at 1 month, defined as Cerebral Performance Category 3–5. The discriminatory ability of bowel diameter was assessed using the area under the receiver operating characteristic curve (AUC).

**Results:**

Data from 181 patients were analyzed (median age, 57 years; 84.5% male). Bowel diameter demonstrated a weak ability to predict unfavorable neurological outcomes, with an AUC of 0.58 (95% confidence interval [CI], 0.47–0.70). However, higher discriminatory performance was observed in subgroups of patients aged ≥ 65 years (AUC, 0.83; 95% CI, 0.69–0.96) and those with initial nonshockable rhythms (AUC, 0.69; 95% CI, 0.47–0.91).

**Conclusion:**

The results of this exploratory investigation suggest that bowel diameter may represent a potential adjunctive marker associated with functional outcomes post‐ECPR for OHCA.

## 1. Introduction

Extracorporeal cardiopulmonary resuscitation (ECPR), a cardiopulmonary resuscitation (CPR) strategy using extracorporeal membrane oxygenation (ECMO), represents a recent advancement in resuscitation science. ECPR provides rapid circulatory support and may improve outcomes post–out‐of‐hospital cardiac arrest (OHCA) [1–5]. Nevertheless, neurological outcomes remain poor, with low favorable recovery rates at hospital discharge (only 11.8%) [6].

Poor neurological prognosis following ECPR is primarily driven by hypoxic brain injury resulting from cerebral hypoperfusion during cardiac arrest and subsequent reperfusion injury [7, 8]. Ischemia–reperfusion affects multiple organ systems; the gastrointestinal tract is particularly susceptible to hypoperfusion. Adenosine triphosphate depletion impairs smooth muscle contractility, causing bowel atony, loss of peristalsis, and progressive intestinal dilation [9, 10]. Reperfusion exacerbates dilation by generating reactive oxygen species, damaging endothelial cells, and triggering inflammation. Capillary leak and interstitial edema contribute to bowel wall thickening, increased intraluminal pressure, and impaired intestinal transit [11, 12]. These pathological changes appear as increased bowel diameter upon imaging, such as computed tomography (CT) [13]. Imaging‐based assessment of bowel diameter may thus reflect systemic ischemia during cardiac arrest and serve as a surrogate marker for cerebral hypoxic injury. Bowel dilation is, however, a nonspecific finding that may result from several potentially coexisting processes—including systemic ischemic burden during no‐flow and low‐flow periods, reperfusion injury, fluid resuscitation, vasopressor exposure, paralytic ileus, and preexisting gastrointestinal conditions—and the present study does not attempt to isolate any single causal pathway. We, therefore, regard bowel diameter as a candidate, nonspecific marker of global ischemic burden rather than a direct mechanistic readout.

As ECPR requires substantial resources [14] and OHCA carries a poor prognosis [6], early prognostication is essential. It enables timely, transparent communication with families and supports shared decision‐making regarding continuation or withdrawal of intensive therapy [15]. Compared with traditional clinical indicators and biomarkers [16, 17], CT offers immediate availability, making it a valuable prognostic tool. However, to the best of our knowledge, no studies have examined the prognostic value of bowel diameter in predicting neurological outcomes post‐ECPR. Intestinal imaging may provide clinicians an accessible, noninvasive indicator of global ischemic burden and improve accuracy and timeliness of outcome prediction.

We aimed to conduct an exploratory investigation on the association between bowel diameter measured on early‐phase abdominal CT and neurological outcomes in patients treated with ECPR for OHCA. This was an exploratory, hypothesis‐generating analysis; we did not aim to establish bowel diameter as a clinically applicable prognostic tool, and any such application would require prospective external validation.

## 2. Materials and Methods

This observational study was conducted using medical records from a tertiary emergency hospital. Approval was obtained from the Institutional Ethics Committee of Metropolitan Bokutoh Hospital (approval number, 07‐029). Requirement for informed consent was waived because data were anonymized before analysis. The study followed the Strengthening the Reporting of Observational Studies in Epidemiology guidelines [18].

### 2.1. Study Setting

Details of resuscitation and intensive care at our institution have been reported previously [19–21]. In brief, indications for ECPR in OHCA include age ≤ 65 years with an initial rhythm of ventricular fibrillation (VF)/ventricular tachycardia (VT) or age ≤ 70 years, collapse after the arrival of emergency medical service personnel, and presumption of a reversible cause. ECMO cannulations were performed percutaneously in a hybrid emergency room equipped with a CT scanner and fluoroscopic imaging, allowing real‐time–guided cannulation. Patients were resuscitated and managed according to the current guideline recommendations [22]. Patients on ECMO underwent targeted temperature management. Core temperature was maintained at 34°C for 24 h, followed by rewarming to 36°C over 12 h. For patients with contraindications (e.g., bleeding, hemodynamic collapse, lethal arrhythmias, or septic shock), the target temperature was 36°C for 24 h.

### 2.2. Study Population

We reviewed institutional medical records from January 1, 2015, through December 31, 2021, to identify patients who underwent ECPR for OHCA. The exclusion criteria were as follows: (1) the absence of abdominal CT before intensive care unit admission and (2) inability to evaluate bowel diameter due to patient‐ or image‐related factors.

### 2.3. Variables and Outcomes

Data on age, sex, etiology of arrest, witness status, bystander CPR frequency, initial rhythm, time from collapse to ECMO, time from collapse to CT scan, time from hospital arrival to CT scan, and time from ECMO to CT scan, prehospital return of spontaneous circulation, radiologic findings, and survival and neurological outcomes at 1 month after the event were extracted from electronic medical records.

Bowel diameter was measured by a single investigator (S.H.) blinded to outcomes, using the picture archiving and communication system (SYNAPSE software, Fujifilm Medical Co., Tokyo, Japan). The most dilated small‐bowel loop was identified on abdominal CT, and the maximal inner wall‐to‐inner wall (luminal) short‐axis diameter of the most dilated small‐bowel loop was measured with electronic calipers at standard abdominal window settings (Figure 1). CT timing was recorded relative to collapse, hospital arrival, and ECMO initiation. The brain CT images obtained on admission and on the following day were independently assessed by radiologists blinded to outcomes for signs of diffuse anoxic injury, defined as loss of gray–white matter differentiation; early head CT is recognized to have limited sensitivity for hypoxic‐ischemic injury.

**FIGURE 1:**
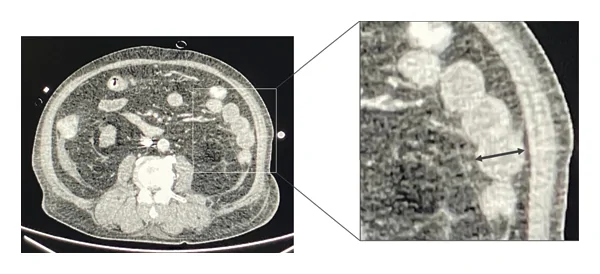
Measurement of bowel diameter on abdominal computed tomography image. The axial slice with the most dilated bowel is selected, and the maximal inner wall‐to‐inner wall (luminal) short‐axis diameter is measured.

The neurological status at 1 month post‐OHCA was determined by the treating physician, using the cerebral performance category (CPC), as follows: Category 1, good cerebral performance; 2, moderate disability; 3, severe disability; 4, coma or vegetative state; and 5, death or brain death. The primary outcome was unfavorable neurological outcomes at 1 month post‐OHCA, defined as CPC of 3–5. The secondary outcome was mortality at 1 month post‐OHCA.

### 2.4. Statistical Analysis

Continuous variables were presented as medians with interquartile ranges or means with standard deviations, depending on distribution. Normality was assessed using the Shapiro–Wilk test. Continuous variables were compared using the Mann–Whitney *U* test or Student’s *t*‐test, as appropriate. Categorical variables were presented as counts and percentages and compared using the chi‐square test.

The discriminatory ability of bowel diameter was assessed using the area under the receiver operating characteristic curve (AUC) and 95% confidence intervals (CIs). Optimal cutoff values were determined using the Youden index, and sensitivity, specificity, positive predictive value (PPV), and negative predictive value (NPV) at the cutoff were calculated.

The associations between bowel diameter dichotomized at the cutoff and neurological outcomes were examined using multivariable logistic regression analyses, adjusted for covariates identified through a directed acyclic graph (Supporting Figure 1). The selected covariates included age, witness status, bystander CPR frequency, initial rhythm, time from collapse to ECMO, time from collapse to CT scan, and presumed etiology. Collinearity was evaluated with variance inflation factors.

The discriminatory abilities of bowel diameter dichotomized at the corresponding cutoff values and anoxic brain injury findings on head CT obtained on admission were compared using the DeLong test. Subgroup analyses were conducted with 1‐month unfavorable neurological outcomes as an outcome in subgroups stratified by age (< 65 or ≥ 65 years), sex, initial rhythm (VF/VT or pulseless electrical activity/asystole), and presumed etiology of the arrest (ischemic heart disease or other).

Analyses were performed using R, Version 4.1.1 (R Foundation for Statistical Computing, Vienna, Austria). Two‐tailed *p* values < 0.05 were considered statistically significant.

## 3. Results

During the study period, 189 patients underwent ECPR for OHCA. After excluding 4 patients without abdominal CT images and 4 whose bowel diameter could not be assessed (because of a massive retroperitoneal hematoma in 1, a large renal cyst in 1, and imaging artifacts in 2 patients), 181 were eligible for analysis (Figure 2). Their median age was 57 years (interquartile range, 48–65), and 153 (84.5%) were male. The first recorded cardiac rhythm was VF/VT in 126 patients (69.6%). At 1 month post‐OHCA, 144 patients (79.6%) exhibited unfavorable neurological outcomes, and 120 (66.3%) had died. Bowel diameter followed a normal distribution, with a mean of 24.6 mm and a standard deviation of 4.8 mm (median, 24.6 mm; Figure 3). CT scan was performed after ECMO establishment and before intensive care unit admission in all evaluable patients, with a median interval from hospital arrival to abdominal CT of 48 min (interquartile range, 30–122 min).

**FIGURE 2:**
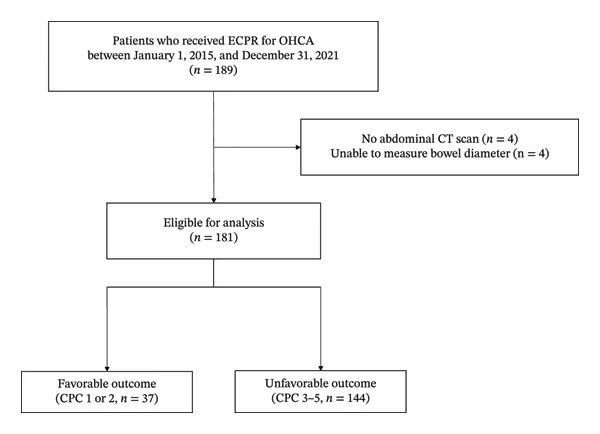
Study flowchart. CPC, cerebral performance category; CT, computed tomography; ECPR, extracorporeal cardiopulmonary resuscitation; OHCA, out‐of‐hospital cardiac arrest.

**FIGURE 3:**
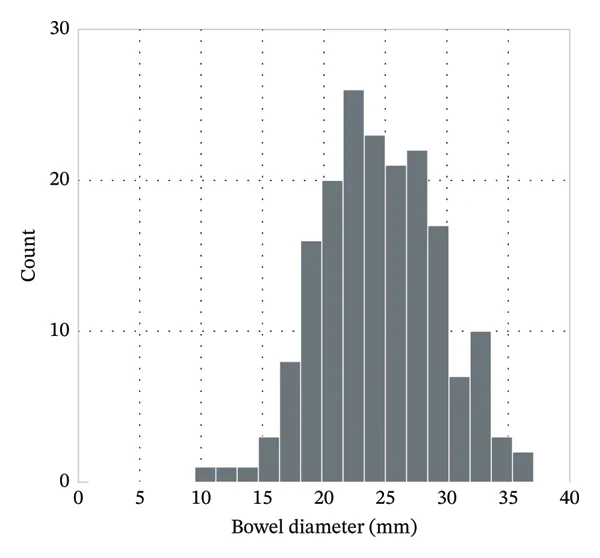
Distribution of bowel diameter. Bowel diameter among the 181 included patients follows a normal distribution, with both the mean and median measuring 24.6 mm and a standard deviation of 4.8 mm.

The baseline characteristics of the patients are shown in Table 1. Bystander CPR frequency was significantly lower among patients with 1‐month unfavorable outcomes compared with those with 1‐month favorable outcomes (68.1% vs. 86.5%, *p* = 0.044). Time from collapse to CT scan (median, 78 vs. 102 min, *p* = 0.045) and time from ECMO to CT scan (median, 30 vs. 64 min, *p* = 0.031) were significantly shorter in patients with 1‐month unfavorable outcomes. Bowel diameter was larger in patients with 1‐month unfavorable outcomes than in those with 1‐month favorable outcomes, although the difference was nonsignificant (mean, 24.9 mm vs. 23.3 mm, *p* = 0.076). Bowel diameter demonstrated a significant association with 1‐month mortality (mean, 25.1 mm vs. 23.5 mm, *p* = 0.035, Supporting Table 1).

**TABLE 1:** Patient characteristics based on 1‐month neurological outcome.

Characteristic	Overall (*n* = 181)	1‐month neurological outcome		*p*‐value	Missing data, *n* (%)
		Unfavorable^†^ (*n* = 144)	Favorable (*n* = 37)		
Age, years, median (IQR)	57 (48, 65)	58 (48, 65)	56 (46, 64)	0.642	0 (0.0)
Age, category, years					
< 65	130 (71.8)	102 (70.8)	28 (75.7)	0.705	
≥ 65	51 (28.2)	42 (29.2)	9 (24.3)		
Male, *n* (%)	153 (84.5)	125 (86.8)	28 (75.7)	0.157	0 (0.0)
Witnessed arrest, *n* (%)	171 (94.5)	136 (94.4)	35 (94.6)	0.999	0 (0.0)
Bystander CPR, *n* (%)	130 (71.8)	98 (68.1)	32 (86.5)	0.044	0 (0.0)
Shockable, *n* (%)	126 (69.6)	100 (69.4)	26 (70.3)	0.999	0 (0.0)
First documented cardiac rhythm, *n* (%)				0.910	0 (0.0)
VF	124 (68.5)	98 (68.1)	26 (70.3)		
VT	2 (1.1)	2 (1.4)	0 (0.0)		
PEA	45 (24.9)	36 (25.0)	9 (24.3)		
Asystole	10 (5.5)	8 (5.6)	2 (5.4)		
Ischemic heart disease etiology, *n* (%)	98 (54.1)	77 (53.5)	21 (56.8)	0.863	0 (0.0)
Time from collapse to ECMO, min, median (IQR)	43 (36, 52)	44 (37, 52)	40 (34, 52)	0.406	2 (1.1)
Time from collapse to CT scan, min, median (IQR)	81 (61, 140)	78 (61, 125)	102 (68, 194)	0.045	3 (1.7)
Time from hospital arrival to CT scan, min, median (IQR)	48 (30, 122)	44 (30, 116)	75 (29, 206)	0.342	0 (0.0)
Time from ECMO to CT scan, min, median (IQR)	34 (19, 96)	30 (19, 74)	64 (23, 122)	0.031	5 (2.8)
Bowel diameter, mm, mean (SD)	24.6 (4.8)	24.9 (4.4)	23.3 (6.1)	0.076	0 (0.0)
Cerebral performance category, *n* (%)					0 (0.0)
1	25 (13.8)	0 (0.0)	25 (67.6)		
2	12 (6.6)	0 (0.0)	12 (32.4)		
3	10 (5.5)	10 (6.9)	0 (0.0)		
4	14 (7.7)	14 (9.7)	0 (0.0)		
5	120 (66.3)	120 (83.3)	0 (0.0)		

*Note:* ECMO, extracorporeal membrane oxygenation; IQR, interquartile range.

Abbreviations: CPR, cardiopulmonary resuscitation; CT, computed tomography; PEA, pulseless electrical activity; SD, standard deviation; VF, ventricular fibrillation; VT, ventricular tachycardia.

^†^Unfavorable neurological outcome was defined as Cerebral Performance Category 3–5.

### 3.1. Prediction of Outcomes Using Bowel Diameter

The AUCs of bowel diameter for predicting 1‐month unfavorable neurological outcomes and 1‐month mortality were 0.58 (95% CI, 0.47–0.70; Figure 4) and 0.59 (95% CI, 0.50–0.68), respectively; the corresponding optimal cutoff values determined by the Youden index were 18.6 and 20.4 mm. At these thresholds, sensitivities were 0.95 and 0.88, specificities were 0.24 and 0.31, PPVs were 0.83 and 0.71, and NPVs were 0.56 and 0.56, respectively. In the multivariable logistic regression, bowel diameter dichotomized by the corresponding cutoff values was independently associated with 1‐month unfavorable neurological outcomes (adjusted odds ratio [aOR], 6.59; 95% CI, 1.99–23.8) and 1‐month mortality (aOR, 3.09; 95% CI, 1.36–7.17). The variance inflation factors were < 1.8, indicating no collinearity among the variables.

**FIGURE 4:**
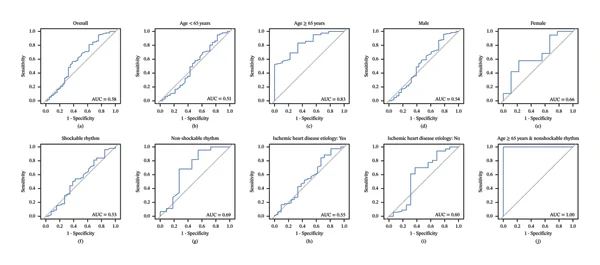
Receiver operating characteristic curves of bowel diameter in predicting 1‐month unfavorable neurological outcomes (cerebral performance category of 3–5). AUC, area under the receiver operating characteristic curve.

Subgroup analyses revealed variation in discriminatory ability by subgroups (Figure 4 and Table 2). The predictive accuracy was the highest in patients aged ≥ 65 years (AUC, 0.83; 95% CI, 0.69–0.96), followed by those with initial nonshockable rhythms (AUC, 0.69; 95% CI, 0.47–0.91). The subgroup of patients aged ≥ 65 years with initial nonshockable rhythms comprised only 16 patients (13 with unfavorable and 3 with favorable neurological outcomes). At the subgroup‐specific cutoff of 19.3 mm, all 13 unfavorable cases had bowel diameters ≥ 19.3 mm, and all 3 favorable cases had diameters < 19.3 mm, resulting in complete (perfect) separation and a nominal AUC of 1.00 (95% CI, 1.00–1.00). This value should be interpreted as an artifact of perfect separation in a very small subgroup rather than as evidence of perfect discrimination and is highly susceptible to overfitting. Within this subgroup, the AUC of bowel diameter was numerically higher than that of anoxic injury on head CT (1.00 vs. 0.54, *p* < 0.001; Table 3).

**TABLE 2:** Discriminatory ability and diagnostic metrics of bowel diameter in predicting 1‐month unfavorable neurological outcomes.

	AUC (95% CI)	Cutoff, mm	Sensitivity	Specificity	PPV	NPV
Overall	0.58 (0.47–0.70)	18.6	0.95	0.24	0.83	0.56
Subgroup analysis						
Age category						
< 65 years	0.51 (0.37–0.65)	18.6	0.95	0.18	0.81	0.50
≥ 65 years	0.83 (0.69–0.96)	24.3	0.52	1.00	1.00	0.31
Sex						
Male	0.54 (0.40–0.67)	18.6	0.96	0.21	0.85	0.55
Female	0.66 (0.43–0.89)	22.0	0.58	0.78	0.85	0.47
First recorded cardiac rhythm						
Shockable	0.53 (0.39–0.67)	20.9	0.81	0.31	0.82	0.30
Nonshockable	0.69 (0.47–0.91)	22.2	0.68	0.73	0.91	0.36
Ischemic heart disease etiology						
Yes	0.55 (0.40–0.70)	20.9	0.84	0.33	0.82	0.37
No	0.60 (0.40–0.79)	22.3	0.70	0.63	0.89	0.33
Age ≥ 65 years and nonshockable rhythm	1.00 (1.00–1.00)	19.3	1.00	1.00	1.00	1.00

*Note:* AUC, area under the receiver operating characteristic curve.

Abbreviations: CI, confidence interval; NPV, negative predictive value; PPV, positive predictive value.

**TABLE 3:** Comparison of discriminatory ability for unfavorable neurological outcome between dichotomized bowel diameter and head CT findings.

	Bowel diameter		Anoxic brain injury on head CT	*p* value
	Cutoff (mm)	AUC (95% CI)	AUC (95% CI)	
Overall	18.6	0.60 (0.53–0.67)	0.60 (0.57–0.63)	0.916
Subgroup analysis				
Age category				
< 65 years	18.6	0.57 (0.49–0.64)	0.63 (0.59–0.67)	0.154
≥ 65 years	24.3	0.77 (0.69–0.85)	0.54 (0.50–0.58)	< 0.001
Sex				
Male	18.6	0.59 (0.51–0.67)	0.60 (0.56–0.63)	0.823
Female	22.0	0.68 (0.50–0.86)	0.63 (0.53–0.73)	0.679
First recorded cardiac rhythm				
Shockable	20.9	0.56 (0.47–0.66)	0.60 (0.56–0.64)	0.542
Nonshockable	22.2	0.71 (0.55–0.86)	0.61 (0.55–0.68)	0.293
Ischemic heart disease etiology				
Yes	20.9	0.59 (0.48–0.71)	0.55 (0.51–0.58)	0.413
No	22.3	0.66 (0.53–0.80)	0.66 (0.61–0.72)	0.990
Age ≥ 65 years and nonshockable rhythm	19.3	1.00 (1.00–1.00)	0.54 (0.46–0.61)	< 0.001

*Note:* AUC, area under the receiver operating characteristic curve.

Abbreviations: CI, confidence interval; CT, computed tomography.

## 4. Discussion

The findings of the present exploratory study suggest that the association between bowel diameter on early‐phase CT and 1‐month unfavorable neurological outcomes post‐OHCA may differ according to patient characteristics. Within the small subgroup of older patients with an initial nonshockable rhythm, the numerically higher discriminatory performance of bowel diameter relative to anoxic injury on head CT should be regarded as an exploratory observation rather than evidence of diagnostic superiority, particularly because early head CT has limited sensitivity for hypoxic‐ischemic injury and the subgroup was very small.

Our results align with prior studies linking gastrointestinal dysfunction—such as ischemic mucosal changes and early diarrhea—with poor neurological outcomes post–cardiac arrest [23, 24]. These observations are supported by studies describing mechanisms of gastrointestinal ischemia, including impaired smooth muscle contractility, oxidative injury, and inflammatory responses [9–12].

The relatively stronger association observed in older patients and those with initial nonshockable rhythms may reflect a greater overall ischemic burden. Older patients are more vulnerable to ischemia because of reduced mesenteric perfusion reserve and impaired vascular autoregulation [25]. Similarly, patients with initial nonshockable rhythms typically experience longer no‐flow or low‐flow periods, compared with those with initial shockable rhythms [26, 27]. These factors may act synergistically, producing more severe bowel dilation, leading to a higher predictive value of bowel diameter. Given the bowel’s sensitivity to hypoxia, the pronounced dilation may reflect the same ischemic burden driving cerebral injury.

Current prognostication post–cardiac arrest relies on neurological examination, biomarkers, electrophysiological testing, and neuroimaging [22]. Each provides valuable information but is limited by cost, invasiveness, or delayed applicability. In contrast, body CT is frequently performed during post‐ECPR management and is readily available. However, the overall discriminatory ability of bowel diameter was modest (AUC approximately 0.58), indicating limited standalone predictive values. Bowel diameter is not proposed to replace or to be added to guideline‐recommended multimodal neuroprognostication performed after 72 h, and any incremental prognostic value beyond established assessments remains unproven. These findings are hypothesis‐generating and would require prospective external validation before any role in clinical decision‐making or family communication could be considered.

This study has some limitations. First, it was conducted at a single center, and self‐fulfilling bias cannot be excluded; external validation is required. Second, the limited sample size, particularly regarding the limited number of older patients and those with an initial nonshockable rhythm included in the study, may have led to overestimation of discriminatory performance due to subgroup‐based analyses; in particular, the AUC of 1.00 observed in patients aged ≥ 65 years with a nonshockable rhythm reflected perfect separation in only 16 patients and should be regarded as overfitting rather than evidence of perfect discrimination. Third, the variability in CT timing may have affected bowel measurements, as intestinal changes evolve postarrest. Fourth, the absence of standardized CT protocols could have influenced measurement accuracy. Fifth, bowel diameter was measured by a single investigator, and intra‐ and interobserver reproducibility (e.g., intraclass correlation coefficients) could not be assessed in this retrospective design; prospective multireader reliability assessment is an essential next step. Sixth, decisions to withdraw life‐sustaining therapy may have introduced a self‐fulfilling prophecy bias, and the timing of multimodal neuroprognostication was not standardized. Seventh, bowel‐related clinical variables (e.g., paralytic ileus or other abdominal pathology) and data on vasopressor exposure and fluid balance were not available, precluding adjustment for these potential contributors to bowel dilation. Eighth, generalizability is limited by the single hybrid‐emergency‐room ECPR system, and no internal or external validation cohort was available. The variation in optimal cutoff values across subgroups (approximately 18–24 mm) further indicates threshold instability. These limitations reinforce that the present findings are exploratory and hypothesis‐generating.

## 5. Conclusion

In the present exploratory investigation, bowel diameter measured on early‐phase CT demonstrated a weak association with neurological outcomes in patients undergoing ECPR for OHCA. Although higher predictive performance was observed in certain patient subgroups, these results are hypothesis‐generating. Further large multicenter studies are needed to establish external validity and evaluate its role in prognostic modeling.

## Funding

The author has nothing to report.

## Conflicts of Interest

The authors declare no conflicts of interest.

## Supporting Information

Additional supporting information can be found online in the Supporting Information section.

## Supporting information


**Supporting Information 1** Supporting Table 1: Patient characteristics based on 1‐month survival.

**Supporting Information 2** Supporting Figure 1: A directed acyclic graph shows potential causal relationships among the variables and the outcome. CPR, cardiopulmonary resuscitation; CT, computed tomography; ECMO, extracorporeal membrane oxygenation.

## Data Availability

The data that support the findings of this study are available from the corresponding author upon reasonable request.
