# Supplementary material for: Early‐Phase Computed Tomography Bowel Diameter and Neurological Outcomes Post‐Extracorporeal Cardiopulmonary Resuscitation
Source: Emerg Med Int. 2026 Oct 9;2026:4291375. doi: 10.1155/emmi/4291375 (PMC13650513; doi:10.1155/emmi/4291375)
Supplement: Supplementary file 1 [file EMMI-2026-4291375-s001.pdf]

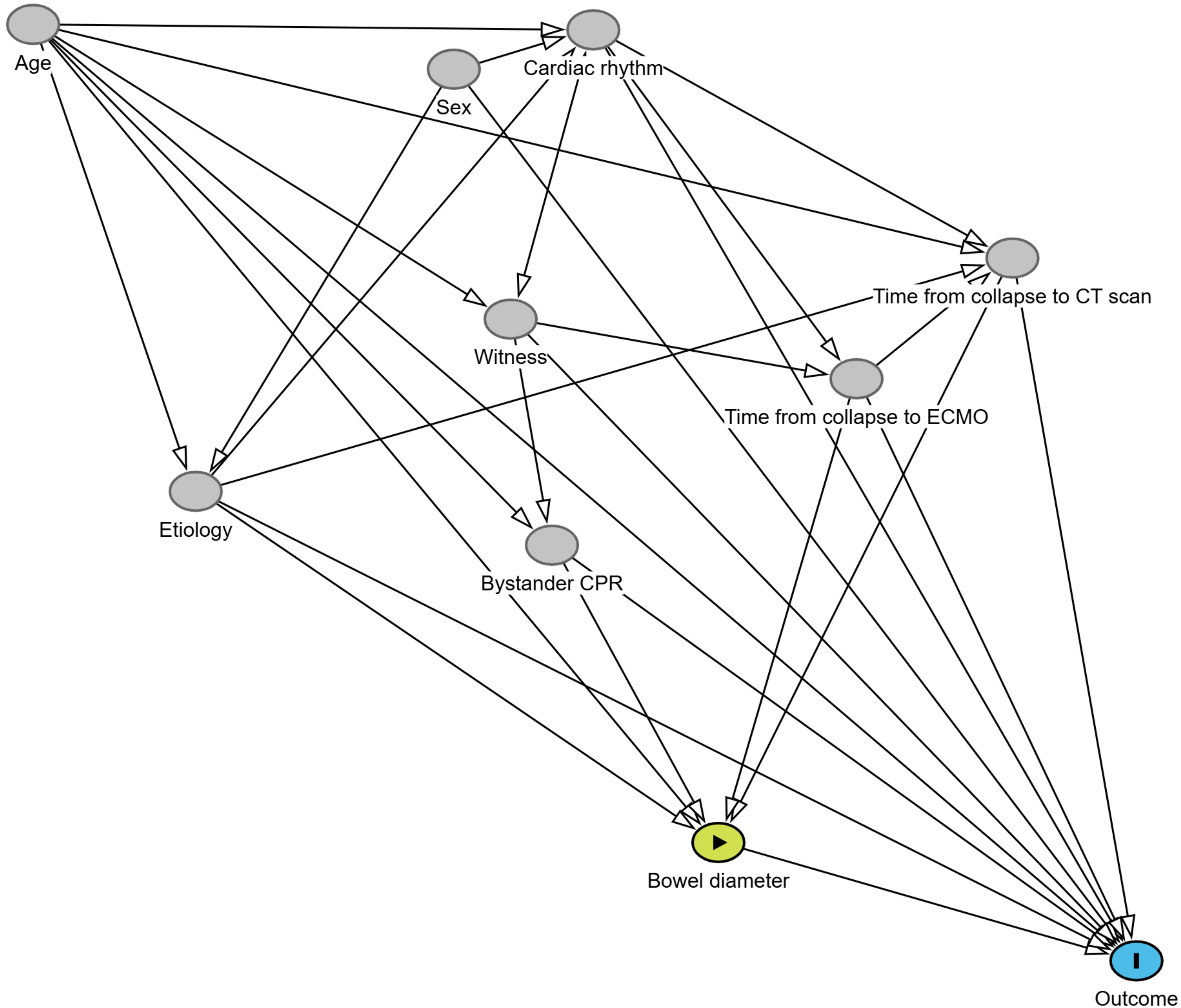

Age
Sex
Cardiac rhythm
Witness
Time from collapse to CT scan
Time from collapse to ECMO
Etiology
Bystander CPR
Bowel diameter
Outcome
