# Supplementary material for: Early‐Phase Computed Tomography Bowel Diameter and Neurological Outcomes Post‐Extracorporeal Cardiopulmonary Resuscitation
Source: Emerg Med Int. 2026 Oct 9;2026:4291375. doi: 10.1155/emmi/4291375 (PMC13650513; doi:10.1155/emmi/4291375)
Supplement: Supplementary file 2 [file EMMI-2026-4291375-s002.docx]

**Supplementary Table 1.** Patient characteristics based on 1-month survival

| Characteristic | Overall  (n = 181) | Death  (n = 120) | Survival  (n = 61) | *P*-value | Missing data,  n (%) |
| --- | --- | --- | --- | --- | --- |
| Age, years, mean (SD) | 55.5 (13.2) | 55.7 (12.6) | 55.0 (14.5) | 0.725 | 0 (0.0) |
| Age, category, years |  |  |  |  |  |
| <65 | 130 (71.8) | 85 (70.8) | 45 (73.8) | 0.810 |  |
| ≥65 | 51 (28.2) | 35 (29.2) | 16 (26.2) |  |  |
| Male, n (%) | 153 (84.5) | 102 (85.0) | 51 (83.6) | 0.978 | 0 (0.0) |
| Witnessed arrest, n (%) | 171 (94.5) | 114 (95.0) | 57 (93.4) | 0.929 | 0 (0.0) |
| Bystander CPR, n (%) | 130 (71.8) | 80 (66.7) | 50 (82.0) | 0.047 | 0 (0.0) |
| Shockable, n (%) | 126 (69.6) | 80 (66.7) | 46 (75.4) | 0.299 | 0 (0.0) |
| First documented cardiac rhythm, n (%) |  |  |  | 0.590 | 0 (0.0) |
| VF | 124 (68.5) | 79 (65.8) | 45 (73.8) |  |  |
| VT | 2 (1.1) | 1 (0.8) | 1 (1.6) |  |  |
| PEA | 45 (24.9) | 32 (26.7) | 13 (21.3) |  |  |
| Asystole | 10 (5.5) | 8 (6.7) | 2 (3.3) |  |  |
| Ischemic heart disease etiology, n (%) | 98 (54.1) | 64 (53.3) | 34 (55.7) | 0.881 | 0 (0.0) |
| Time from collapse to ECMO, min, median (IQR) | 43 (36, 52) | 45 (38, 52) | 39 (33, 51) | 0.038 | 2 (1.1) |
| Time from collapse to CT scan, min, median (IQR) | 81 (61, 140) | 78 (61, 122) | 97 (64, 185) | 0.053 | 3 (1.7) |
| Time from hospital arrival to CT scan, min, median (IQR) | 48 (30, 122) | 44 (32, 111) | 67 (27, 195) | 0.451 | 0 (0.0) |
| Time from ECMO to CT scan, min, median (IQR) | 34 (19, 96) | 28 (19, 69) | 59 (22, 126) | 0.012 | 5 (2.8) |
| Bowel diameter, mm, mean (SD) | 24.6 (4.8) | 25.1 (4.3) | 23.5 (5.6) | 0.035 | 0 (0.0) |
| Cerebral Performance Category, n (%) |  |  |  |  | 0 (0.0) |
| 1 | 25 (13.8) | 0 (0.0) | 25 (41.0) |  |  |
| 2 | 12 (6.6) | 0 (0.0) | 12 (19.7) |  |  |
| 3 | 10 (5.5) | 0 (0.0) | 10 (16.4) |  |  |
| 4 | 14 (7.7) | 0 (0.0) | 14 (23.0) |  |  |
| 5 | 120 (66.3) | 120 (100.0) | 0 (0.0) |  |  |

CPR, cardiopulmonary resuscitation; CT, computed tomography; ECMO, extracorporeal membrane oxygenation; IQR, interquartile range; PEA, pulseless electrical activity; SD, standard deviation; VF, ventricular fibrillation; VT, ventricular tachycardia.
